# Association of Catechol-O-methyltransferase (COMT Val^158^Met) with future risk of cardiovascular disease in depressed individuals - a Swedish population-based cohort study

**DOI:** 10.1186/s12881-018-0645-2

**Published:** 2018-07-25

**Authors:** Aysha Almas, Yvonne Forsell, Vincent Millischer, Jette Möller, Catharina Lavebratt

**Affiliations:** 1 Department of Public Health Sciences, Karolinska Institutet, 171 77 Stockholm, Sweden; 2 Department of Medicine, Aga Khan University, Karachi, Pakistan; 3 Department of Molecular Medicine and Surgery, Karolinska Institutet, Stockholm, Sweden; 4 Neurogenetics Unit, Center for Molecular Medicine, Karolinska University Hospital, L8:00, 171 76 Stockholm, Sweden

**Keywords:** Genetic variation, Depression, Myocardial infarction, Stroke, Gender

## Abstract

**Background:**

Catechol-O-methyltransferase (COMT Val^158^Met) has been implicated in both depression and cardiovascular disease. The purpose of this study was to assess if COMT Val^158^Met, which influences the COMT enzyme activity, has an effect on the risk of cardiovascular disease (CVD) in individuals with a history of depression and also to determine if the risk differs depending on gender.

**Methods:**

Data from a longitudinal cohort study of mental health among Swedish adults was used. Depression was assessed twice 3 years apart for each participant, in 1998–2001 and 2001–2003. Saliva DNA was contributed by 4349 (41.7%) of the participants and 3525 was successfully genotyped for COMT Val^158^Met. Participants were followed up until December 2014 from the National Patient register with regard to cardiovascular outcomes (hypertensive or ischemic heart disease, and stroke).

**Results:**

Those with depression and the high COMT enzyme activity genotype (Val/Val) had almost a three-fold increased risk of later CVD (OR 3.6; 95% CI: 2.0-6.6) compared to those non-depressed carrying the Val/Val allele. This effect on risk for CVD was higher in women compared to men (OR 7.0; 95% CI: 3.0-14.0 versus OR 2.1; 95% CI: 1.0-6.8). Both additive interaction (attributable proportion (AP) = 0.56; 95% CI: 0.24-0.90 and synergy index (SI) = 4.39; 1.0-18.7) and multiplicative interaction (log likelihood test *p* = 0.1) was present between depression and COMT Val^158^Met in predicting risk of later CVD.

**Conclusion:**

High COMT activity genotype Val^158^Met increased the risk of CVD in depressed persons. The risk was higher in women compared to men.

## Background

Epidemiological and family studies have repeatedly shown that genetic predisposition accounts for 40–60% of the risk for coronary artery disease. Correspondingly for depression, twin studies suggest a heritability of 40–50%, and family studies indicate a two- to threefold increase in lifetime risk of developing depression among first-degree relatives [1]. Multiple studies have shown that depression is a risk factor for cardiovascular diseases (CVD) including coronary heart disease and stroke [2]. Thus genetic vulnerability is important in both CVD and depression, and some of these genetic underpinnings may be shared between the disorders.

Catechol-O-methyltransferase (COMT) has previously been implicated in both depression and CVD. The enzyme COMT is expressed in several tissues and degrades not only dopamine but also other catecholamines and sex steroids, like catechol estrogens and dietary polyphenols. Animal and human studies have shown that altered levels of dopamine neurotransmission contribute to depressive-like behavior and influence depressive symptoms [3, 4]. Dopamine, [5] catechol amines [6, 7] and estrogens [8] have well-known effects on the cardiovascular system, e.g. blood pressure regulation. The COMT enzymatic activity is dependent on genetic variations in the *COMT* gene. The Val^158^Met has a large effect on the enzymatic activity and the minor allele is quite frequent in many human populations. COMT Val^158^Met is a substitution of methionine (Met) for valine (Val) at codon 158 encoded by a single nucleotide polymorphism (SNP), rs4680. The Met allele has a lower enzymatic activity compared to the Val allele. The Val/Val genotype is associated with approximately 40% more effective degradation of dopamine compared to the Met/Met genotype, while those with Val/Met genotype display an intermediate COMT activity [9, 10].

Although COMT Val^158^Met has not shown significance in genome-wide association studies (GWAS) on depression, a recent meta-analysis by Wang et al. suggested an effect on major depressive disorder depending on ethnicity, with Val being the vulnerability allele in Europeans [11, 12]. The COMT Val^158^Met has also been reported to be associated with cardiovascular disease and metabolic disorder. COMT Val^158^Met homozygosity for the low-activity allele (Met/Met), has been associated with myocardial infarction (n_cases_ = 69, n_controls_ = 723) [13] and metabolic disorders like abdominal obesity and high blood pressure in men (*n* = 240) [14]. In contrast, in a larger cohort study in Swedes by Eriksson et al. (n_cases_ = 174, n_controls_ = 348), Met/Met was reported to be protective against myocardial infarction [8]. The purpose of this study was to determine the effect of COMT Val^158^Met on the risk of CVD among depressed persons. Based on the fact that the Val allele was the risk allele for depression in the meta analysis in Europeans [11], and Met/Met homozygosity had a protective effect on myocardial infarction in the large Swedish cohort [14], we hypothesized that the Val allele might increase the risk for depression leading to CVD. Because of previous gender-specific associations for COMT Val^158^Met with depression and CVD [14–16] we performed gender-stratified analyses.

## Methods

### Cohort

This project utilized data from the PART study (In Swedish short for: Psykisk hälsa, Arbete och RelaTioner), a longitudinal cohort study of mental health, work and relations among randomly selected adults (20–64 years) residing in Stockholm County, Sweden. The Ethical Review Board at Karolinska Institutet, Stockholm, approved the study (case number: 96–260, 97–313, 01–218, 03–302, 2004–528/3, 2009/880–31, 2012/808–32. After a complete description of the study to the subjects, written informed consent was obtained. The PART study had three measurement points: wave 1 (W1) in 1998–2000, wave 2 (W2) in 2001–2003 and wave 3 (W3) in 2010. At each wave, participants answered a postal questionnaire. The questionnaire was divided into two parts, the first one comprised questions about childhood conditions, socioeconomic and demographic factors, coping-strategies, financial status, working conditions, social network, life events, somatic disorders and use of medication. The second part included screening instruments for psychological wellbeing and psychiatric symptoms.

**Fig. 1:**
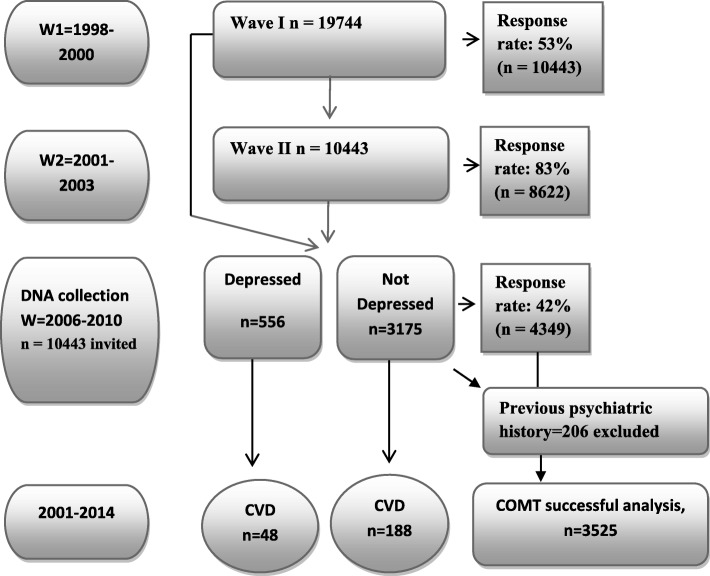
Patient Recruitment for DNA collection from the PART study.

The PART study aimed to include 19,744 persons out of which 19,457 could be reached, and 10,443 individuals responded to the questionnaire at W1 (participation rate 53%). Non-response analyses were performed using available administrative registers, and participation was related to female gender, higher age, higher income and education, being born in the Nordic countries and having no previous psychiatric diagnosis in inpatient registers [17]. In the following two waves the participation rates were 83% (*n* = 8622) and 61% (*n* = 5228). Attrition in W2 was associated with similar factors as in W1 [18]. All respondents in W1 (*n* = 10,443) were invited to provide saliva for DNA and 4349 (42%) participated and were followed up for occurrence of cardiovascular disease event between 2001 and 2014 in the National Patient Register (NPR) [19] (Figure 1). Those with previous psychiatric illness were excluded from the non-depressed group (*n* = 206).

### Definition of depression

A participant was assessed as ‘Depressed’ if scored with depression in W1 or W2 or both waves according to the Major Depression Inventory (MDI) [20]. The MDI has shown high validity in both clinical and non-clinical samples [21, 22]. The MDI scale comprises 10 questions on symptoms present nearly every day during the past 2 weeks. Each question has five response alternatives scored from 1 to 5 according to the presence of the symptom; all the time (5), most of the time (4), slightly more than half of the time (3), slightly less than half of the time (2) some of the time (1) and never (0). The sum score of all 10 questions ranges from 0 to 50. In both W1 and W2 of PART, a subsample was interviewed by psychiatrists using Schedules for Clinical Assessment in Neuropsychiatry to validate the MDI scale for diagnosis of depression. Using an MDI score cutoff > 20, the sensitivity was 78% and the specificity was 73% for Major depressive disorder, and 67 and 79%, respectively, for all depressive disorders [23]. In this current study we used cutoff MDI > 20 for defining depression.

### Definition of cardiovascular disease

Cardiovascular disease (CVD) was assessed by hospital discharge diagnoses from the Swedish National Patient Register (NPR) between 2001 and 2014 [24]. The following diagnoses according to the international classification of diseases (ICD10) were used and were grouped together as cardiovascular diseases: ischemic/hypertensive heart disease; hypertensive diseases (I11-I13), ischemic heart diseases (I20-I25), heart failure (I50), other peripheral vascular diseases, embolism and thrombosis (I73-I74); and stroke (I60-I67 and I69).

### DNA sampling and genotyping

In 2006–2007 and 2010–2011, all participants who had responded in the first wave (W1) were invited to contribute DNA using a self-administered whole-saliva DNA sample collection kit (Oragene, DNA Genotek Inc., Ottawa, Canada) sent to their homes. Saliva was obtained from 4349 (42%) participants and genomic DNA was extracted using Oragene Purifier. The COMT Val^158^Met (rs4680) genotype was successfully obtained for 3731 samples (91% of the randomly selected 4107 samples) using TaqMan SNP genotyping assays applying an ABI 7900 HT instrument (Applied Bio systems, Foster City, CA) [15]. Of 1443 samples run in duplicate plates, 96% had successful and identical result in both plates.

### Statistical analyses

Logistic regression was used to calculate odds ratios (OR) and corresponding 95% confidence intervals (95% CI) for depression and CVD given COMT Val^158^Met, adjusting for age and body mass index (BMI). To determine the combined effect of Val^158^Met and depression on later CVD, similar logistic regression analyses were performed using the four dummy variables; Met carriers (A/A plus A/G) with no depression (reference), Met carriers (A/A plus A/G) with depression, Val/Val (G/G) with no depression, and Val/Val (G/G) with depression. Additive interaction was estimated [25] by calculating the following indices [26, 27]: (i) the relative excess risk due to interaction (RERI), (ii) the attributable proportion due to interaction (AP) and (iii) the synergy index (S). RERI is the excess risk due to interaction relative to the risk without exposure. AP refers to the attributable proportion of disease that is due to interaction among individuals with both exposures. S is the excess risk from both exposures when the additive interaction, relative to the risk from both exposures without interaction. RERI ≠ 0, AP ≠ 0, or S ≠ 1 are indicative of additive interaction [28]. Indices results over the null value indicate synergistic interactions; indices below the null value indicate antagonistic interactions [26]. Multiplicative interaction was estimated using a main effect model (depression and COMT Val^158^Met as exposure) with and without multiplicative interaction term between depression and COMT Val^158^Met. The relative goodness of fit among models was established by the Loglikelihood test using the main effect model as reference. A *p*-value of 0.05 was considered to be statistically significant for the main effects; and a p-value of 0.10 was considered to be statistically significant for interaction terms and interaction indices, since epidemiologic data have limited power to detect product terms [29, 30]. SPSS versions 19.11 and SAS 9.3 were used for the statistical analyses.

## Results

Out of the 3525 participants with COMT Val^158^Met data 1094 (31.0%) had Met/Met genotype, 1720 (48.0%) were Met/Val and 711 (20.2%) were Val/Val (Table 1).

**Table 1:** Distribution of COMT Val^158^Met, depression and cardiovascular disease (CVD), stratified by gender

	All (*n* = 3525)	Men (*n* = 1495)	Women (*n* = 2030)
	n (%)		
Depression	556 (15.8)	157 (10.5)	399 (19.7)
Cardiovascular disease	236 (6.7)	152 (10.2)	84 (4.1)
COMT Val ^158^ Met^a^			
Met/Met	1094 (31.0)	457 (30.6)	637 (31.4)
Met/Val	1720 (48.0)	722 (48.3)	998 (49.2)
Val/Val	711 (20.2)	316 (21.1)	395 (19.5)
	Median (25th, 75th percentile)		
Age [years]	46 (34, 55)	47 (35, 55)	45 (33, 54)
BMI [kg/m2]^b^	24.5 (22.5, 26.9)	25.3 (23.4, 27.4)	23.8 (22.0, 26.5)

^a^Met/Met (A/A), Met/Val (A/G), Val/Val (G/G)

^b^BMI: Body mass index

The genotype distribution was in Hardy Weinberg equilibrium (*p* = 0.31). Those homozygous for Val/Val showed a borderline reduced risk for depression (OR = 0.70 (95% CI: 0.60-1.0), Table 2). However, those who were Val/Val had a point-wise increased risk for future CVD (OR = 1.3 (95% CI: 1.0-1.7)). Stratification on gender showed that the OR point estimate for risk for later CVD was higher among women than men (OR = 1.5 (95% CI: 0.8-2.4) and OR = 1.1 (95% CI: 0.7-1.7), respectively, Table 2).

**Table 2:** Association of COMT Val^158^Met with depression and cardiovascular disease; stratified by gender

COMT Val^158^Met	Depression			Cardiovascular disease (CVD)		
	All *n* = 556	Men *n* = 157	Women *n* = 399	All *n* = 236	Men *n* = 152	Womenn = 84
	n _Depressed_/n _Non-depressed_			n _CVD_/n _Non-CVD_		
Met/Met or Met/Val	463/2351	130/1049	333/1302	179/2635	117/1062	62/1573
Val/Val	93/618	27/289	66/329	57/654	35/281	22/373
	Odds ratio (95% confidence interval)^a^					
Met/Met or Met/Val	1 (ref)	1 (ref)	1 (ref)	1 (ref)	1 (ref)	1 (ref)
Val/Val	0.70 (0.60-1.0)	0.74 (0.50-1.1)	0.78 (0.60-1.0)	1.3 (1.0-1.7)	1.1 (0.7-1.7)	1.5 (0.8-2.4)
*p value*	0.02	0.20	0.11	0.13	0.60	0.13

^a^Odds ratio (OR) for Val/Val (G/G) was assessed relative to the reference: Met/Met (A/A) plus Met/Val (A/G), adjusted for age and body mass index

Also, depression had a main effect increasing the risk for CVD in this cohort (OR = 1.9 (95% CI 1.4-2.5)) [31]. Considering both depression and Val^158^Met genotype for future risk of CVD, the OR was 3.6 (95% CI: 2.0-6.6)) for those having both Val/Val and depression and 1.1 (95% CI: 0.8-1.6) for those having Val/Val and no depression (Table 3). The OR (95% CI) for those who were Met carriers and had depression was 1.5 (1.0-2.3). We also stratified the data by gender and found that the point-wise effect on risk of later CVD was higher in women compared to men among those having both Val/Val and depression; OR 7.0 (3.0-14) and 2.1 (1.0-6.8), respectively (Table 3). To explore the possibility of a dilution effect by having Met/Met plus Val/Met in the reference group, we calculated the OR for having Val/Val and depression using the reference group being those having Met/Met and no depression. This OR was 4.2 (95% CI 2.1-8.4) for men and women together, and 8.5 (95% CI 3.4-21.2) for women only. This indicated a slight but no major dilution effect by including both Met/Met and Met/Val in the reference group (corresponding ORs being 3.6 and 7.0, respectively, Table 3).

**Table 3:** Interaction between COMT Val^158^Met and depression for later cardiovascular disease (CVD), stratified by gender

	All (n = 3525)		Men (n = 1495)		Women (n = 2030)	
	Met/Met or Met/Val	Val/Val	Met/Met or Met/Val	Val/Val	Met/Met or Met/Val	Val/Val
	*n* = 2814	*n* = 711	*n* = 1179	*n* = 316	*n* = 1635	*n* = 395
Depression	n _CVD_/n _Non-CVD_					
No	146 /2205	42/576	102/947	31/258	44/1258	11/318
Yes	33/430	15/78	15/115	4/23	18/315	11/55
	Odds ratio (95% confidence interval)^a^					
Depression						
No	1 (Ref)	1.1 (0.8 1.6)	1 (Ref)	1.1 (0.73 1.7)	1 (Ref)	1.0 (0.50 2.0)
*P values*	–	0.5	–	0.5	–	0.10
Yes	1.5 (1.0-2.3)	3.6 (2.0-6.6)	1.8 (1.0-3.4)	2.1 (1.0 6.8)	2.0 (1.1 3.5)	7.0 (3.0 14.0)
*P values*	0.03	< 0.001	0.05	0.20	0.01	< 0.001

^a^Odds ratio (OR) for Val/Val (G/G) with no depression, Val/Val (G/G) with depression, and Met carriers (A/A plus A/G) with depression, adjusted for age and body mass index. Met carriers (A/A plus A/G) with no depression was the reference group

Indices of additive interaction in the sample demonstrating additive interaction between depression and Val^158^Met genotype for later CVD are shown in Table 4. For multiplicative interaction, effect size of the interaction term and the loglikelihood test comparing the main effect model and the model with interaction term are shown in Table 4 and indicate borderline statistical significance.

**Table 4:** Additive and multiplicative interaction analyses between COMT Val^158^Met and depression for later cardiovascular disease (CVD) (*n* = 3525)

Interaction indices	Estimate (95% CI)
Additive interaction^a^	
RERI	2.06 (− 0.22-4.3)
AP	0.56 (0.24-0.88)
S	4.39 (1.0-18.7)
Multiplicative interaction	Odds ratio (95% CI)
*Model 1 - main effects*	
Depression (yes)	1.4 (1.0-2.0)
Val/Val	1.3 (1.0-1.8)
*Model 2 – main and interaction effects*	
Depression (yes)	1.2 (0.70-1.7)
Val/Val	1.1 (0.80-1.6)
Depression x Val^158^Met	2.2 (1.0-4.7)
P-value (Model 2 versus Model 1)	0.10^b^

^a^RERI: the relative excess risk due to interaction

*AP* the attributable proportion due to interaction

*S* the synergy index

AP > 0 and S > 1 indicate additive interaction

^b^Log likelihood test (− 2 log likelihood: Model 1 = 1725.6; Model 2 = 1721.2)

## Discussion

Depression is a known risk factor for CVD [31–33]. The COMT Val^158^Met genetic variation influencing COMT enzyme activity has previously been associated with risk for depression [12], and risk for CVD [8, 13, 14]. The identity of the at risk allele has varied between studies, although a meta-analysis demonstrated high activity *COMT* Val allele as risk allele for depression. An influence of gender as well as childhood adversity on the Val^158^Met association with depression has previously been reported [15], although a recent meta-analysis found no Val^158^Met association to depression in any gender [16]. Using a large population-based Swedish cohort of adults we here show for the first time that the COMT Val^158^Met genotype, corresponding to high COMT enzymatic activity, implies an increased risk of CVD especially for those who had depression up to 14 years earlier. Thus, both an additive and a multiplicative interaction between depression and COMT Val^158^Met for risk of CVD were detected. Additionally, this risk of CVD by high COMT activity genotype and depression was more pronounced in women compared to men. Both mild and severe depression were considered, scored at two time points for each participant, and the original cohort was randomly selected among Swedish nationals in the Stockholm County.

There are previous reports demonstrating a relationship between COMT Val^158^Met and acute coronary events, ischemic stroke and CVD risk factors like hypertension and lipid abnormalities [13, 14, 34]. The results from these studies are however not fully consistent with regard to which allele implies a disease risk and the influence of depression on the relationship was not previously assessed. Hagen and coworkers reported that high COMT activity (Val/Val genotype) is overrepresented in male and female Norwegians with systolic hypertension (≥140 mmHg) (*n* = 2591) [34]. This finding was confirmed in a Chinese population (*n* = 3079) showing that high activity COMT (Val/Val) was associated with cardio -metabolic risk factors including hypertension and high triglyceride levels [35]. Accordingly, Eriksson et al. reported a protective effect of low activity COMT (Met/Met or Val/Met) against myocardial infarction in Swedish and Finnish hypertensive men (*n* = 522) [8]. Contrary to this, low activity COMT (Met/Met or Val/Met) was associated with acute coronary events in Finnish men (*n* = 792) [13], and with high systolic and diastolic blood pressure and abdominal obesity in Swedish men (*n* = 1302) [14]. The reason for the discrepancy in risk allele identity between the aforementioned studies is unclear but could in part be related to different ranges of estrogen levels, and thereby different gender and age distributions. Accordingly, we found that high activity *COMT* (Val/Val)*depression was associated with increased CVD risk in women, but not in men. This sex-based difference might partially be explained by the difference in estrogen activity between men and women. Estrogen plays an important role in the cardiovascular system and COMT is key in the degradation of estrogens. Thus, the association between COMT Val/Val and CVD in females might reflect altered levels of estrogen and its metabolites [8, 36]. Moreover, estrogen signaling influences *COMT* transcription through estrogen response elements in the *COMT* promoter [37, 38]. The COMT enzyme metabolizes also dopamine and catecholamines which regulate both mood and cardiovascular functions through wide-spread expression of their receptors. Therefore, our Val/Val-CVD association finding may partly be due to effects of COMT enzyme activity variation on the metabolism of these transmitters. The influence of depression on the Val/Val-CVD association may in part be through increased inflammation and oxidative stress often seen in the depressed state, [39] which could potentiate a high COMT enzyme activity effect on cardiovascular function. Of the individuals in PART 11% had a non-Swedish origin, among those the vast majority had a Nordic origin. The Swedish population at time of sampling had no strong internal genetic borders [40] and especially the southern/middle parts of Sweden (from where the participants of this study are derived) were more genetically homogeneous [41].

### Limitations

Firstly, due to the self-administered sampling at home, the depression cases that participated did likely not represent those most severely depressed. Secondly, only 42% provided DNA samples. Factors associated with public refusal to consent to DNA biobanking in the PART have been reported and reveal that, a lack of personal relevance of DNA contribution and feelings of discomfort related to the DNA being used for purposes other than the respective study were the reasons for low participation [42] The association between depression and risk of later CVD is unlikely influenced by refusal to consent to DNA biobanking. Another limitation of the study is that we did not have individual data on psychotropic drugs medication. Antipsychotic drugs are known to increase risk for CVD [43]. Also, we did not include data from the cause of death register and the outpatient register, hence we might have missed those who died or visited outpatient department due to IHD or stroke without prior hospitalizations.

## Conclusion

The risk for later CVD was increased in depressed persons with high activity COMT Val^158^Met genotype (Val/Val), with a synergistic interaction between depression status and *COMT* genotype. This effect on risk for CVD was higher in women and might in part reflect estrogen signaling. The findings warrant further studies.

## Abbreviations

CVD: Cardiovascular diseases
HTN: Hypertension
IHD: Ischemic heart Diseases
MDI: Major depression inventory
